# Supplementary material for: Hsa-mir-135a Shows Potential as A Putative Diagnostic Biomarker in Saliva and Plasma for Endometriosis
Source: Biomolecules. 2022 Aug 19;12(8):1144. doi: 10.3390/biom12081144 (PMC9405570; doi:10.3390/biom12081144)
Supplement: Supplementary file 1 [file biomolecules-12-01144-s001.zip › biomolecules-1815083-supplementary.pdf]

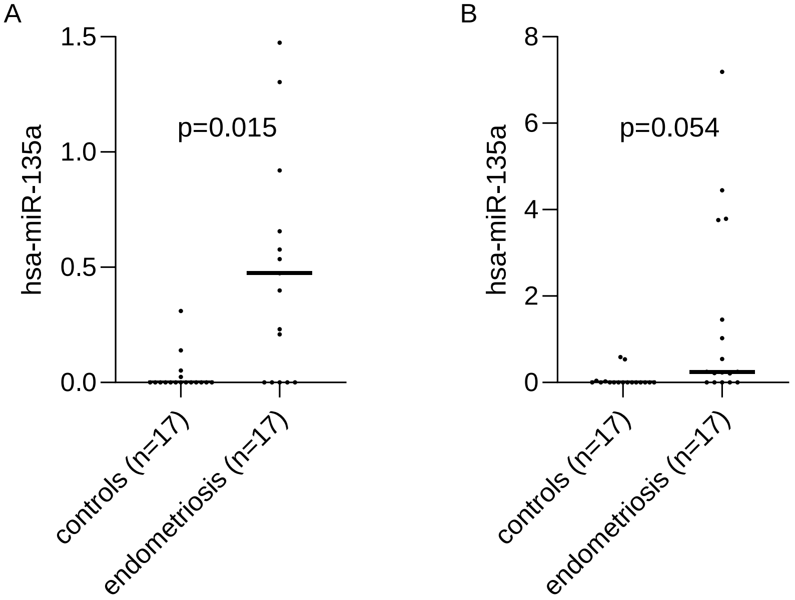

Supplementary Figure S1. Alternative normalization methods confirm that hsa-miR-135a expression is upregulated in endometriosis patients. (**A**) Firefly custom multiplex miRNA expression data was normalized to the arithmetic mean of the 28 miRNAs plus the spike-in miRNA.
(**B**) Firefly custom multiplex miRNA expression data was normalized to the non-standard geometric mean of the 28 miRNAs plus the spike-in miRNA. The non-standard geometric mean was calculated after the removal of zero values as described by Habib (2012) previously [36]. Significant differences between the groups were detected by a Mann–Whitney-U-test followed by correction for multiple testing applying Bonferroni-Holm algorithms. The adjusted p-value is shown on the graph.

Supplemetary Table S1A. Results after testing the distribution of the levels of miRNAs within the samples are shown. The analysis was done using SPSS based Kolmogorov-Smirnov and Shapiro-Wilk methods. The correction of the data was performed using Lilliefors correction method.

normality test type	Kolmogorov-Smirnov			Shapiro-Wilk		
**miRNA**	**statistic**	**dF**	**significance**	**statistic**	**dF**	**significance[a]**
hsa-mir-16-5p	0,335	34	0,00000	0,555	34	0,00000
hsa-mir-191-5p	0,314	34	0,00000	0,538	34	0,00000
hsa-mir-195-5p	0,343	34	0,00000	0,449	34	0,00000
hsa-mir-362-5p	0,274	34	0,00000	0,698	34	0,00000
hsa-mir-135a	0,296	34	0,00000	0,728	34	0,00000
hsa-mir-17-5p	0,334	34	0,00000	0,462	34	0,00000
hsa-mir-20a-5p	0,349	34	0,00000	0,442	34	0,00000
hsa-mir-22-3p	0,301	34	0,00000	0,515	34	0,00000
hsa-mir-141-3p	0,37	34	0,00000	0,361	34	0,00000
hsa-mir-200a-3p	0,37	34	0,00000	0,357	34	0,00000
hsa-mir-145-5p	0,278	34	0,00000	0,642	34	0,00000
hsa-mir-31-5p	0,356	34	0,00000	0,424	34	0,00000
hsa-mir-154-5p	0,365	34	0,00000	0,536	34	0,00000
hsa-mir-378a-3p	0,326	34	0,00000	0,473	34	0,00000
hsa-mir-196b-5p	0,376	34	0,00000	0,336	34	0,00000
hsa-mir-33a-5p	0,405	34	0,00000	0,347	34	0,00000
hsa-let-7d-5p	0,326	34	0,00000	0,559	34	0,00000
hsa-mir-145-3p	0,456	34	0,00000	0,48	34	0,00000
hsa-mir-584	0,366	34	0,00000	0,445	34	0,00000
hsa-mir-9-3p	0,357	34	0,00000	0,504	34	0,00000
hsa-mir-141-5p	0,393	34	0,00000	0,386	34	0,00000
hsa-mir-122-5p	0,329	34	0,00000	0,642	34	0,00000
hsa-mir-103a-3p	0,341	34	0,00000	0,449	34	0,00000
hsa-mir-30e-5p	0,258	34	0,00000	0,626	34	0,00000
hsa-mir-126-5p	0,322	34	0,00000	0,615	34	0,00000
hsa-mir-93-5p	0,322	34	0,00000	0,501	34	0,00000
hsa-mir-92a-3p	0,378	34	0,00000	0,402	34	0,00000

a: Lilliefors correction metod
dF: degree of freedom

Supplemetary Table S1B. Results variability test of the levels of miRNAs are shown. The analysis was performed using SPSS.

miRNAs	samples number	MFI	SD	CV
hsa-mir-362-5p	34	1,037	1,516	1,462
hsa-mir-135aCorr	34	0,196	0,287	1,470
hsa-mir-30e-5pCorr	34	0,142	0,219	1,542
hsa-mir-122-5pCorr	34	0,703	1,191	1,695
hsa-mir-145-5pCorr	34	5,667	9,645	1,702
hsa-mir-126-5pCorr	34	0,106	0,198	1,865
hsa-mir-22-3pCorr	34	674,976	1293,144	1,916
hsa-mir-16-5pCorr	34	1202,317	2392,011	1,990
hsa-let-7d-5pCorr	34	37,644	75,327	2,001
hsa-mir-191-5pCorr	34	355,427	729,532	2,053
hsa-mir-154-5pCorr	34	0,062	0,132	2,136
hsa-mir-93-5pCorr	34	267,012	578,367	2,166
hsa-mir-378a-3pCorr	34	14,680	32,632	2,223
hsa-mir-9-3pCorr	34	0,287	0,657	2,285
hsa-mir-17-5pCorr	34	228,531	534,547	2,339
hsa-mir-15b-5pCorr	34	249,505	589,568	2,363
hsa-mir-145-3pCorr	34	0,028	0,068	2,388
hsa-mir-103a-3pCorr	34	225,891	552,404	2,445
hsa-mir-195-5pCorr	34	55,454	137,303	2,476
hsa-mir-20a-5pCorr	34	157,505	392,185	2,490
hsa-mir-584Corr	34	0,424	1,056	2,492
hsa-mir-31-5pCorr	34	6,904	18,257	2,645
hsa-mir-92a-3pCorr	34	150,574	398,268	2,645
hsa-mir-141-5pCorr	34	0,986	2,870	2,911
hsa-mir-141-3pCorr	34	32,091	96,410	3,004
hsa-mir-200a-3pCorr	34	50,667	153,180	3,023
hsa-mir-33a-5pCorr	34	1,986	6,067	3,055
hsa-mir-196b-5pCorr	34	0,247	0,780	3,164

MFI, Mean Fluorescence Intensity; SD, Standard deviation; CV, coefficient of variation

Supplementary Table S2. Expression profile of focused panel miRNAs in saliva of women with and without endometriosis

**miRNA**	**n= not expressed (MFI=0)**	**n=expressed (MFI>0)**	**total sample size**	**% below detection level**
hsa-mir-16-5p	0	34	34	0
hsa-mir-191-5p	0	34	34	0
hsa-mir-22-3p	1	33	34	2,94
hsa-mir-141-3p	1	33	34	2,94
hsa-mir-17-5p	2	32	34	5,88
hsa-mir-20a-5p	2	32	34	5,88
hsa-mir-15b-5p	3	31	34	8,82
hsa-mir-362-5p	3	31	34	8,82
hsa-mir-200a-3p	3	31	34	8,82
hsa-mir-33a-5p	3	31	34	8,82
hsa-mir-92a-3p	3	31	34	8,82
hsa-mir-195-5p	4	30	34	11,76
hsa-mir-103a-3p	4	30	34	11,76
hsa-mir-93-5p	6	28	34	17,65
hsa-mir-378a-3p	8	26	34	23,53
hsa-let-7d-5p	9	25	34	26,47
hsa-mir-141-5p	11	23	34	32,35
hsa-mir-30e-5p	12	22	34	35,29
hsa-mir-31-5p	13	21	34	38,24
hsa-mir-122-5p	15	19	34	44,12
hsa-mir-145-5p	16	18	34	47,06
hsa-mir-9-3p	16	18	34	47,06
hsa-mir-584	17	17	34	50,00
hsa-mir-135a	18	16	34	52,94
hsa-mir-196b-5p	18	16	34	52,94
hsa-mir-154-5p	21	13	34	61,76
hsa-mir-126-5p	21	13	34	61,76
hsa-mir-145-3p	27	7	34	79,41

Supplementary Table S3. Differentially expressed miRNAs in saliva of women with endometriosis compared to women without endometriosis.

miRNA	exact p-value	adj.p-value (Bonferoni-Holm)
hsa-mir-135a	0,0008	0,022568
hsa-mir-126-5p	0,0080	0,216
hsa-mir-196b-5p	0,0097	0,252434
hsa-mir-584	0,0155	0,388175
hsa-mir-141-5p	0,0215	0,515976
hsa-mir-145-3p	0,0296	0,68034
hsa-mir-9-3p	0,0317	0,697708
hsa-mir-122-5p	0,066	1,386
hsa-mir-195-5p	0,128	2,56
hsa-mir-30e-5p	0,129	2,451
hsa-mir-378a-3p	0,154	2,772
hsa-mir-362-5p	0,245	4,165
hsa-mir-22-3p	0,259	4,144
hsa-mir-92a-3p	0,306	4,59
hsa-mir-145-5p	0,307	4,298
hsa-mir-17-5p	0,318	4,134
hsa-mir-33a-5p	0,322	3,864
hsa-mir-93-5p	0,369	4,059
hsa-mir-191-5p	0,394	3,94
hsa-mir-15b-5p	0,413	3,717
hsa-mir-16-5p	0,454	3,632
hsa-mir-31-5p	0,461	3,227
hsa-mir-103a-3p	0,479	2,874
hsa-mir-141-3p	0,496	2,48
hsa-mir-154-5p	0,506	2,024
hsa-let-7d-5p	0,680	2,04
hsa-mir-20a-5p	0,805	1,61
hsa-mir-200a-3p	1,000	28
